# Recent Breakthroughs in the Antioxidant and Anti-Inflammatory Effects of *Morella* and *Myrica* Species

**DOI:** 10.3390/ijms160817160

**Published:** 2015-07-28

**Authors:** Bruno J. C. Silva, Ana M. L. Seca, Maria do Carmo Barreto, Diana C. G. A. Pinto

**Affiliations:** 1 Department of Technological Science and Development, University of Azores, Ponta Delgada 9501-801, Portugal; E-Mails: 20137026@aluno.uac.pt (B.J.C.S.); anaseca@uac.pt (A.M.L.S.); barreto@uac.pt (M.C.B.); 2 Department of Chemistry & Química Orgânica Produtos Naturais and Agroalimentares, University of Aveiro, Aveiro 3810-193, Portugal; 3 Centro Investigação Recursos Naturais, University of Azores, Ponta Delgada 9501-801, Portugal

**Keywords:** *Morella*, *Myrica*, antioxidant, anti-inflammatory, flavonoids, diarylheptanoid

## Abstract

Oxidative stress is one of the risk factors for the development of several chronic diseases, such as diabetes, cancer, cardiovascular and neurodegenerative diseases. Antioxidants are therefore highly sought and can be seen as a type of preventive medicine against several diseases. *Myrica* and *Morella* genus (Myricaceae) are taxonomically very close and their species are trees or shrubs with edible fruits that exhibit relevant uses in traditional medicine, for instance in Chinese or Japanese folk medicine they are used to treat diarrhea, digestive problems, headache, burns and skin diseases. Nearly 36 compounds were isolated from different morphological parts of *Myrica* and/or *Morella* species and their antioxidant and anti-inflammatory activities evaluated. Thirteen of these compounds exhibit greater effects than the positive controls used. Adenodimerin A was the most active compound reported (in a 2,2-diphenyl-1-picrylhydrazyl (DPPH) assay EC_50_= 7.9 ± 0.3 µM). These results are just one aspect of the antioxidant and anti-inflammatory evaluations reported regarding *Myrica* and *Morella* species, so a comprehensive overview on the current status, highlighting the antioxidant health promoting effect of these species, their key antioxidant compounds as well as the compounds with protective effects against oxidative stress related diseases such as inflammation, is relevant.

## 1. Introduction

Pro-oxidants can be of endogenous or exogenous origin and lead to oxidative stress by creating reactive oxygen species (ROS) such as superoxide radical anion (O_2_^●^^−^), hydroxyl radical (OH^●^), peroxyl radical (ROO^●^) and hydrogen peroxide (H_2_O_2_), and/or reactive nitrogen species (RNS) such as nitric oxide radical (NO^●^) and peroxynitrite (ONOO^−^) [1,2]. ROS and RNS oxidize or nitrosylate proteins, unsaturated lipids, micronutrients and DNA (deoxyribonucleic acid) [3]. When at low levels, ROS and RNS have important physiological functions, such as in disulphide bond formation [4] and in a myriad of cellular signalling pathways [4,5]. For example, a recent review emphasizes the importance of these species in redox signalling pathways that mediate the immune response [6]. However, pathological oxidative stress arises when their concentrations exceed the maximum output of the cellular antioxidant enzyme systems (CAT (catalase), GPx (glutathione peroxidase), GR (glutathione reductase), Trx (thioredoxin), TR (thioredoxin reductase) and SOD (superoxide dismutase)), and other systems like small antioxidant molecules and apoptotic/repair mechanisms [4,7]. Due to the unspecific targets of ROS and RNS, it is unsurprising that a high levels of oxidative stress has been associated with increased risk for developing several chronic diseases in humans, such as chronic inflammatory response [8,9] cardiovascular [10,11], autoimmune and neurodegenerative diseases [12,13], cancer [9] and diabetes [14]. It is also responsible for faster aging due to telomere shortening [15], male infertility [16] and disuse muscle atrophy [17].

Antioxidants reduce oxidative stress and therefore play an essential role in improving well-being, preventing many of the pathological conditions listed above [7,14,16,18,19]. Antioxidant activity is usually related to direct scavenging of ROS and RNS. But it can also be related to inhibition of the enzymes that produce ROS and RNS, or the inhibition of NF-kB (nuclear factor kappa β) and the stimulation of Nrf-2 (nuclear factor erythroid 2-related factor 2, a regulator of cellular resistance to oxidants) [20]. Antioxidants can be used as functional foods, as dietary supplements, in complementary medicine systems and as additives in foodstuffs to maintain flavour [21,22]. Although they are generally harmless and have a massive importance in disease prevention, recent studies demonstrated that they can release acrylamide, a neurotoxin and carcinogen [23]. So care should be taken with their use because they can also be harmful [24].

In recent years secondary metabolites isolated and/or obtained by synthesis are considered more reliable [25] so the search for antioxidant secondary metabolites is a hot topic in the natural products research field. In this context, we will present the most recent developments on natural antioxidants isolated from *Myrica* and *Morella* species.

## 2. *Myrica* and *Morella* Genera

The *Myrica* genus belongs to the Myricaceae family and, before 2002 comprised about 97 species widely distributed by both temperate and sub-tropical regions [26,27]. Macdonald *et al.* [28] stated reasons for dismembering this genus in two, *Myrica* and *Morella*, but only in 2002 his arguments were accepted and the genus was split [29]. To clarify the distinction a taxonomic key was published in 2005 [30]. Thus, many of the species previously belonging to the *Myrica* genus have been reclassified and are now in the *Morella* genus. This taxonomic reclassification has several consequences in the natural products research field. Several studies published before 2005 report the isolation of secondary metabolites from the *Myrica* species, which are now *Morella* species, and this can cause misleading reports on secondary metabolites found for the first time in the genus. But it can also result in more recent publications that still use the previous scientific name, and consequently not be detected properly in a literature survey. Thus, we decided in this work, to join both genera and report their secondary metabolites to which antioxidant activities were endorsed.

*Morella* is by far the largest genus, having about 50 described species with a wide distribution in North America, Europe, Africa, and Asia [30]. All *Myrica* and *Morella* species are woody shrubs or tree pioneers in nitrogen-poor soils such as sandy soil or gravelly sites, because they are actinorhizal plants, and exhibit the ability to fix nitrogen through nitrogen-fixing root nodules induced by soil actinomycetes of the genus *Frankia*, with which it establishes a symbiotic relationship [31]. In addition to the economic interest of these species as source of paper and rope from the bark, as fuel wood, for biomass production and land reclamation, they are also appreciated because of their fruits that can be eaten raw, used in the production of jams, syrups and juices [27] and their applications in traditional medicine are also noteworthy. Indeed, several species of these genera are used as medicines in countries with relevant traditional medicine systems (see Table 1). Moreover, very important to remember, traditional medicine is either the mainstay of health care delivery or serves as its complement in many countries and the demand for its services is currently increasing [32].

**Table 1:** Ethnopharmacological uses and distribution of *Myrica* and *Morella* species.

Species Name ^a^	Distribution	Traditional Uses
*Morella*		
*Morella adenophora* (Hance) J. Herb.	China and Taiwan	Roots and bark to treat bleeding, diarrhea and stomach pain [33].
*Morella nana* (A. Chev.) J. Herb.	China	Fruits are beneficial for dyspepsia [34]. Roots are used to treat bleeding, diarrhea, stomach pain, burns, and skin diseases [35]. Bark is used to treat enteritis [36].
*Morella serrata* (Lam.) Killick	South Africa and Southern African countries extending into tropical Africa	Used to treat asthma, coughing and shortness of breath [37]. The decoction of the root is used to treat painful menstruation, cold, coughs and headaches and to enhance male sexual performance [38]. It is also used in the management of sugar related disorder and as laxative to treat constipation. The stem bark is used to treat headache [39].
*Morella arborea* (Hutch.) Cheek	Cameroon	Bark decoction used to treat fevers and inflammation [40].
*Morella cerifera* (L.) Small	North America	Herb decoction or tincture used as astringent, diaphoretic, as a circulatory stimulant, to treat irritable bowel syndrome, ulcerative colitis, digestive system disorders, diarrhea, dysentery, leukorrhea, mucous colitis, colds, stomatitis, sore throat, measles and scarlet fever, convulsions, nasal catarrh and jaundice [41].
*Morella salicifolia* (Hochst. ex A.Rich.) Verdc. & Polhill	Southeast Africa, Ethiopia and Saudi Arabia	Roots infusion is used to treat gastro-intestinal disorder [42] while roots and bark used in the treatment of headache [43], pain, inflammation and respiratory diseases [44].
*Myrica*		
*Myrica rubra* (Lour.) Siebold & Zucc.	China, Japan, Taiwan and Korea	The various organs are used to treat gastrointestinal diseases, headaches, burns and skin diseases. Leaves are used to treat inflammatory diseases [45].
*Myrica esculenta* Buch.- Ham. ex D. Don	India, South China, Malaysia, Japan, Vietnam and Nepal	Ayuverdic medicine use decoction of bark to treat asthma, bronchitis, fever, lung infection, dysentery, toothache and wounds [46–48]; leaf, root, bark and fruits juice for worms, jaundice and dysentery [48]; Vietnamese folk medicine uses bark to treat catarrhal fever, cough, sore throat and skin disease [49].
*Myrica gale* L.	Europe, Siberia, Canada and Northern USA	Used in the treatment of ulcers, intestinal worms, cardiac disorders and aching muscles [50].
*Myrica nagi* Thunb.	China, Malaya Islands, Pakistan and Nepal	Bark finds its application in reducing inflammations [51] to treat cardiac diseases, bronchitis, gonorrhea, diuresis, dysentery, epilepsy, gargle, heamoptysis, hypothermia, catarrh, headache, menorrhagia, putrid sores, typhoid, face palsy and paralysis and wounds [51,52]. Fruit wax or oil is used for treating ulcers [53], bleeding piles, body ache, toothache and for regulating the menstrual cycle [52].

^a^, Accepted name as indicated by The International Plant Names Index (IPNI) database.

Several of the traditional applications presented in Table 1, like the use of the oil from the flowers of *Myrica* species to treat inflammation, ear-ache and paralysis [27], are related to their potential to act on numerous oxidative stress effects being an important source of antioxidant and anti-inflammatory medicines.

This potential unleashed the necessary and almost obligatory research to try to prove the beneficial effects suggested by traditional medicine as well as to look for the active ingredients responsible for the activities displayed. Herein are reviewed the most significant studies on the antioxidant and anti-inflammatory compounds isolated from species of the *Morella* and *Myrica* genera.

## 3. Isolated Compounds from *Morella*/*Myrica* Species

A revision of the literature published in the last 15 years, showed 116 compounds isolated from *Myrica*/*Morella* species, mostly cyclic diarylheptanoids, flavonoids and pentacyclic triterpenoids. It bears mentioning that there was little cross-species phytochemical variability to the point where some authors suggest that some cyclic diarylheptanoids, specially, myricanone **1** and myricanol **10**, and some pentacyclic triterpenoids should be used as *Myrica*/*Morella* genus chemotaxonomic markers [54], while unusual *C*-methylated dihydrochalcones and flavonoids may support the segregation of some *Myrica* species to a new genus [55].

Several new diarylheptanoids (e.g., in Table 2), which have a unique 1,7-diphenylheptane structure and are distributed in a few botanic genera [56], have been isolated over the years from the *Myrica* genus(e.g., *Myrica rubra* [45]). Diarylheptanoids are known for their remarkable anti-inflammatory, antioxidant, antitumor, estrogenic, leishmanicidal, melanogenesis, hepatoprotective and neuroprotective activities [56,57].

**Table 2:** Compounds isolated from *Myrica* and *Morella* species with antioxidant and anti-inflammatory activities.

Compound Name (Number)	Chemical Structure	Current Species Name ^a^, Part of Plant
*Diarylheptanoids*		
Myricanone (**1**)	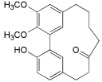	*Mo. adenophora* (Hance) J. Herb., roots [58]; *Mo. arborea* (Hutch.) Cheek, twigs [54]; *Mo. nana* (A. Chev.) J. Herb, roots [34]; *Mo. cerifera* (L.) Small, bark [59], twigs [60]; *My. gale* L. (syn. *My. gale *var. *tormentosa* L.), branches [61];* My. rubra* (Lour.) Siebold & Zucc., bark [62]
5-Deoxymyricanone (**2**)	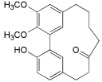	*Mo. adenophora* (Hance) J. Herb*.*, roots [58]
Myricananin C (**3**)	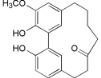	*Mo. adenophora* (Hance) J. Herb*.*, roots [58]; *Mo. nana* (A. Chev.) J. Herb*.*, roots [63]
12-Hydroxymyricanone (**4**)	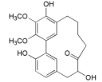	*Mo. adenophora* (Hance) J. Herb*.*, roots [58]; *Mo. nana* (A. Chev.) J. Herb, roots [63]; *My. gale *L. (syn. *My.* *gale *var* tormentosa *L.), branches [61]
Porson ^b^ (**5**)	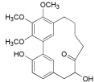	*Mo. adenophora* (Hance) J. Herb*.*, roots [58]; *Mo. nana *(A. Chev.) J. Herb, roots [34]; *My. gale *L. (syn. *My. gale *var* tormentosa *L.), branches [61]
Myricananin D (**6**)	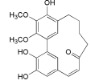	*Mo. adenophora* (Hance) J. Herb., [58]; *Mo. nana* (A. Chev.) J. Herb, roots [63]
Alnusonol (**7**)	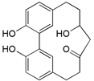	*Mo. nana* (A. Chev.) J. Herb, roots [63]
Actinidione (**8**)	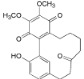	*Mo. adenophora* (Hance) J. Herb*.*, roots [58]; *Mo. nana* (A. Chev.) J. Herb, roots [63]
Galeon (**9**)	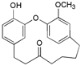	*Mo. adenophora* (Hance) J. Herb*.*, roots [58]; *My. gale *L. (syn. *My.* *gale *var* tormentosa *L.), branches [64]
Myricanol (**10**)	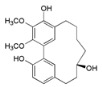	*Mo. adenophora* (Hance) J. Herb., roots [58]; *Mo. arborea* (Hutch.) Cheek, root and stem barks [40], twigs [54]; *Mo. cerifera* (L.) Small, bark [59], root-bark [65]; *My. esculenta* Buch.-Ham. ex D.Don, leaves [66]; *Mo. nana* (A. Chev.) J. Herb, roots [34]; *My. rubra *(Lour.) Siebold & Zucc., bark [62]
Myricanol 11-*O*-β-d-xylopyranoside (**11**)	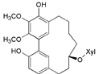	*Mo. adenophora* (Hance) J. Herb*.,* roots [58]; *Mo. arborea *(Hutch.) Cheek, root and stem barks [40]
Myricanol 11-*O*-β-d-glucopyranoside (**12**)	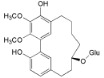	*Mo. adenophora* (Hance) J. Herb., roots [58]; * Mo. nana* (A. Chev.) J. Herb, roots [35]; *My. rubra* (Lour.) Siebold & Zucc., bark [62]
Myricanol 5-*O*-β-d-glucopyranoside (**13**)	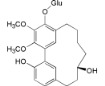	*My. rubra* (Lour.) Siebold & Zucc., bark [62]
Myricanol 5-*O*-β-d-(6′-*O*-galloyl)-glucopyranoside (**14**)	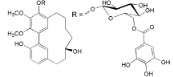	*My. rubra* (Lour.) Siebold & Zucc., bark [62]
Myricananin A (**15**)	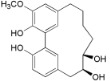	*Mo. nana* (A. Chev.) J. Herb, roots [63]
Juglanin B-11( *R*)-*O*-sulphate (**16**)	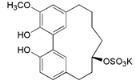	*My. rubra* (Lour.) Siebold & Zucc., leaves [67]
*Flavonoids*		
Myricetin 3-*O*-(2-*O*-galloyl)-α-l-rhamnopyranoside (**17**)	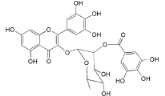	*My. rubra* (Lour.) Siebold & Zucc., leaves [68]
Myricetin 3-*O*-(2-*O*-galloyl)-β-d-galactopyranoside (**18**)	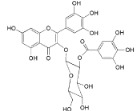	*My. rubra* (Lour.) Siebold & Zucc., leaves [68]
Quercetin 3-*O*-(2-*O*-galloyl)-β-d-galactopyranoside (**19**)	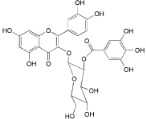	*My. rubra* (Lour.) Siebold & Zucc., leaves [68]
Myricetin (**20**)	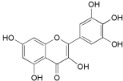	*Mo. adenophora *(Hance) J. Herb., roots [58]; *My. rubra* (Lour.) Siebold & Zucc., leaves [68], bark [62]; *Mo. cerifera* (L.) Small, root-bark [65]; *My. esculenta* Buch.- Ham. ex D.Don, leaves [66]
Myricetin-3′-*O*-sulfate (**21**)	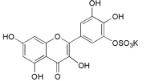	*My. rubra* (Lour.) Siebold & Zucc., leaves [67]
Ampelopsin 3′-*O*-sulfate (**22**)	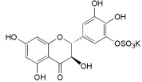	*My. rubra* (Lour.) Siebold & Zucc., leaves [67]
Myricitrin (**23**)	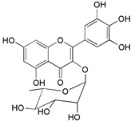	*Mo. adenophora* (Hance) J. Herb., roots [58]; *My. rubra* (Lour.) Siebold & Zucc., leaves [68], bark [62]; *Mo. cerifera* (L.) Small, root-bark [65]; *My. esculenta* Buch.- Ham. ex D.Don, leaves [66]
Quercitrin (**24**)	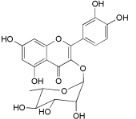	*Mo. adenophora* (Hance) J. Herb*.*, roots [58]
Adenodimerin A (**25**)	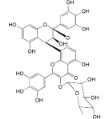	*Mo. adenophora* (Hance) J. Herb*.*, roots [58]
Myricitrin (**23**)	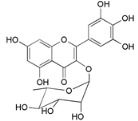	*Mo. adenophora *(Hance) J. Herb., roots [58]; *My. rubra* (Lour.) Siebold & Zucc., leaves [68], bark [62]; *Mo. cerifera* (L.) Small, root-bark [65]; *My. esculenta* Buch.- Ham. ex D.Don, leaves [66]
Procyanidin B2 (**26**)	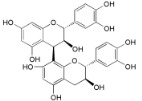	*My. rubra* (Lour.) Siebold & Zucc., fruit pulp [69]
(−)-Epicathechin (**27**)	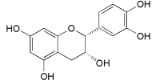	*My. rubra* (Lour.) Siebold & Zucc., fruit pulp [69];* My. gale* L., aerial parts [70]
Cyanidin 3-*O*-glucopyranoside (**28**)	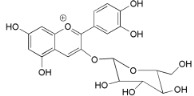	*My. rubra* (Lour.) Siebold & Zucc., fruits [45]
*Miscellaneous Compounds*		
Myricalactone (**29**)	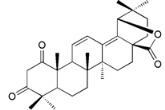	*Mo. adenophora* (Hance) J. Herb*.*, roots [58]; *My. gale *L. (syn. *My. gale *var* tormentosa *L.), stem [64]
3β-*Trans*-*p*-coumaroyloxy-2α,23-dihydroxyolean-12-en-28-oic acid (**30**)	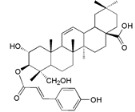	*Mo. adenophora* (Hance) J. Herb*.*, roots [58]
Rhoiptelenol (**31**)	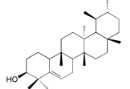	*My. rubra* (Lour.) Siebold&Zucc., bark [71]
Ursolic acid (**32**)	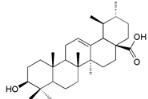	*My. rubra* (Lour.) Siebold&Zucc., bark [71]
β-Sitosterol (**33**)	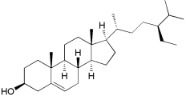	*Mo. adenophora* (Hance) J. Herb*.*, roots [58]; *My. esculenta *Buch.- Ham. ex D.Don, leaves [72]
6′-*O*-galloyl orbicularin (**34**)	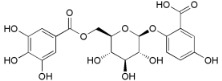	*Mo. adenophora* (Hance) J. Herb*.*, roots [58]
Myricadenin A (**35**)	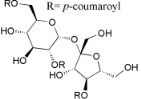	*Mo. adenophora* (Hance) J. Herb*.*, roots [58]
Myricadenin B (**36**)	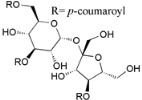	*Mo. adenophora* (Hance) J. Herb*.*, roots [58]
6′-*O*-galloyl orbicularin (**34**)	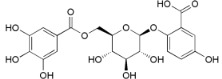	*Mo. adenophora* (Hance) J. Herb*.*, roots [58]

^a^, Always that the botanical name used by the authors is not the presently accepted name following the IPNI database, we report here the correct name where *Mo*. means *Morella *and *My*. means* Myrica*; and ^b^, The original chemical structure was proposed for the first time in 1975 [73] but later was corrected to the one here depicted [61].

Flavonoids are common plant secondary metabolites (e.g., in Table 2), well known for having several biological activities *in vitro* and *in vivo* among which we can highlight their remarkable antioxidant activity, leading to lower chronic disease development [74]. However, some are pro-oxidants as structures with more hydroxyl groups actually lead to an increase in ROS by the Fenton reaction rather than antioxidant activity [74]. Flavonoids also have interesting anti-inflammatory activities against several markers *in vitro*, but *in vivo* studies are still lacking [75].

Pentacyclic triterpenoids are widespread through the plant kingdom and oleanane-type structures are commonplace [76]. *Morella* and *Myrica* genera proved to be no exception. Several activities and molecular targets are associated with this compound type, including antioxidant and anti-inflammatory [77].

From the 116 compounds isolated from *Myrica*/*Morella* species, about a third were evaluated from the point of view of antioxidant and/or anti-inflammatory activities and from these, only 36 compounds were considered in Table 2. Our choice was based on their higher activity and/or on those that allowed structure/activity relationship establishments. And those results will be discussed in the following points of this review.

One important conclusion that arises from Table 2 analysis is that, although *Myrica* and *Morella* genera have several plants with edible fruits with medicinal applications (Table 1) most antioxidant and anti-inflammatory studies were done with secondary metabolites isolated from roots, bark and leaves (Table 2). Nevertheless, finding beneficial antioxidant effects in these species and assessing their usefulness as food supplements, additives or as coadjutants in certain treatments which imply severe ROS/RNS imbalance, not only enhances market interest in their exploration, but also allows the customer to know the health-promoting effects that they might have.

## 4. Biological Activities

### 4.1. Antioxidant Activity

Antioxidant activity is considered a key mechanism to prevent many diseases [78,79,80], including inflammation, diabetes, cancer and cardiovascular diseases. In fact some antioxidants are considered important nutraceuticals due to their beneficial health effects.

Antioxidant activity assays are common and diverse; in recent remarks 407 variants of 29 different *in vitro* and *in vivo* methods for assaying antioxidant capacity were discussed [78]. These different approaches give rise to important issues involving the use of different positive controls, different reagent concentrations and reaction times [80]. Consequently comparisons are almost impossible and to disclose the quality of the reported results is very difficult.

In this work, we decided to highlight and discuss the results (Table 3) obtained by the most common *in vivo* and *in vitro* methods whose trials were performed according to good practice suggestions [80] and that were considered trustworthy (*i.e.*, that exhibit statistical treatment and use a positive control) in order to show the antioxidant potential of several compounds isolated from *Myrica* and *Morella* species. Not only we want to highlight the antioxidant potential of these secondary metabolites but also to discuss some weaknesses and/or strengths of the used methods, and in doing so we expect to stimulate our readers to be more careful and critical in their research methodologies.

**Table 3:** Antioxidant activity of the isolated compounds from *Myrica* and *Morella* species.

Compound	Antioxidant Activity (Positive Control Used)	
1	DPPH: EC_50_ = 202.7 ± 15.8 µM (Ascorbic acid EC_50_ = 22.25 ± 0.4 µM) [58] ABTS: EC_50_ = 19.6 ± 0.7 µM (Ascorbic acid EC_50_ = 23.3 ± 0.2 µM) [58]
2	DPPH: EC_50_ ≥ 250 µM (Ascorbic acid EC_50_ = 22.25 ± 0.4 µM) [58] ABTS: EC_50_ = 102.7 ± 12.4 µM (Ascorbic acid EC_50_ = 23.3 ± 0.2 µM) [58]
3	DPPH: EC_50_ = 16.3 ± 2.8 µM (Ascorbic acid EC_50_ = 22.25 ± 0.4 µM) [58] ABTS: EC_50_ = 12.0 ± 0.6 µM (Ascorbic acid EC_50_ = 23.3 ± 0.2 µM) [58]
5	DPPH: EC_50_ > 250 µM (Ascorbic acid EC_50_ = 22.25 ± 0.4 µM) [58] ABTS: EC_50_ = 73.7 ± 0.1 µM (Ascorbic acid EC_50_ = 23.3 ± 0.2 µM) [58]
6	DPPH: EC_50_ = 87.8 ± 0.0 µM (Ascorbic acid EC_50_ = 22.25 ± 0.4 µM) [58] EC_50_ = 14.9 µM (α-Tocopherol EC_50_ = 27.1 µM) [62] ABTS: EC_50_ = 85.9 ± 2.7 µM (Ascorbic acid EC_50_ = 23.3 ± 0.2 µM) [58]
8	DPPH: EC_50_ = 195.4 ± 2.2 µM (Ascorbic acid EC_50_ = 22.25 ± 0.4 µM) [58] ABTS: EC_50_ = 89.1 ± 0.6 µM (Ascorbic acid EC_50_ = 23.3 ± 0.2 µM) [58]
9	DPPH: EC_50_ = 51.1 ± 2.9 µM (Ascorbic acid EC_50_ = 22.25 ± 0.4 µM) [58] ABTS: EC_50_ = 26.8 ± 1.6 µM (Ascorbic acid EC_50_ = 23.3 ± 0.2 µM) [58]
10	DPPH: EC_50_ = 198.9 ± 9.1 µM (Ascorbic acid EC_50_ = 22.25 ± 0.4 µM) [58] ABTS: EC_50_ = 22.3 ± 0.6 µM (Ascorbic acid EC_50_ = 23.3 ± 0.2 µM) [58]
11	DPPH: EC_50_ = 81.6 ± 3.7 µM (Ascorbic acid EC_50_ = 22.25 ± 0.4 µM) [58] ABTS: EC_50_ = 25.3 ± 2.6 µM (Ascorbic acid EC_50_ = 23.3 ± 0.2 µM) [58]
12	DPPH: EC_50_ > 250 µM (Ascorbic acid EC_50_ = 22.25 ± 0.4 µM) [58] EC_50_ = 12.9 µM (α-Tocopherol EC_50_ = 27.1 µM) [62] ABTS: EC_50_ = 19.6 ± 0.2 µM (Ascorbic acid EC_50_ = 23.3 ± 0.2 µM) [58]
13	DPPH: EC_50_ > 100 µM (α-Tocopherol EC_50_ = 27.1 µM) [62]
14	DPPH: EC_50_ = 6.8 µM (α-Tocopherol EC_50_ = 27.1 µM) [62]
17	NBT: EC_50_ = 0.48 ± 0.02 µM (Allopurinol EC_50_ = 1.23 ± 0.22 µM) [68]
18	NBT: EC_50_ = 0.67 ± 0.03 µM (Allopurinol EC_50_ = 1.23 ± 0.22 µM) [68]
19	NBT: EC_50_ = 1.57 ± 0.30 µM (Allopurinol EC_50_ = 1.23 ± 0.22 µM) [68]
20	DPPH: EC_50_ = 15.9 ± 0.0 µM (Ascorbic acid EC_50_ = 22.25 ± 0.4 µM) [58] EC_50_ = 2.0 µM (α-Tocopherol EC_50_ = 27.1 µM) [62] ABTS: EC_50_ = 15.6 ± 1.4 µM (Ascorbic acid EC_50_ = 23.3 ± 0.2 µM) [58] NBT: EC_50_ = 7.40 ± 0.24 µM (Allopurinol EC_50_ = 1.23 ± 0.22 µM) [68]
23	DPPH: EC_50_ = 2.2 µM (α-Tocopherol EC_50_ = 27.1 µM) [62] NBT: EC_50_ = 5.17 ± 0.23 µM (Allopurinol EC_50_ = 1.23 ± 0.22 µM) [68] Significantly inhibits acrylamide mediated ROS generation and cytotoxicity in Caco-2 cells (*p* < 0.05) at concentrations ranging from 5.4–21.6 µM (2.5–10 µg/mL) [81]. Significantly attenuated intracellular ROS production at 0.1–10 µM and inhibits lipid peroxidation in brain mitochondria (EC_50_ = 3.19 ± 0.34 µM) [82]
25	DPPH: EC_50_ = 7.9 ± 0.3 µM (Ascorbic acid EC_50_ = 22.25 ± 0.4 µM) [58] ABTS: EC_50_ = 7.5 ± 0.4 µM (Ascorbic acid EC_50_ = 23.3 ± 0.2 µM) [58]
26	DPPH: EC_50_ = 3.6 µM (BHA EC_50_ = 14.2 µM) ^a^ [69]
27	DPPH: EC_50_ = 9.8 µM (BHA EC_50_ = 14.2 µM) ^a^ [69]
28	DPPH activity is directly correlated with its concentration [45]
29	DPPH: EC_50_ > 250 µM (Ascorbic acid EC_50_ = 22.25 ± 0.4 µM) [58] ABTS: EC_50_ = 41.9 ± 0.6 µM (Ascorbic acid EC_50_ = 23.3 ± 0.2 µM) [58]
30	DPPH: EC_50_ > 250 µM (Ascorbic acid EC_50_ = 22.25 ± 0.4 µM) [58] ABTS: EC_50_ > 200 µM (Ascorbic acid EC_50_ = 23.3 ± 0.2 µM) [58]
34	DPPH: EC_50_ > 250 µM (Ascorbic acid EC_50_ = 23.3 ± 0.2 µM) [58] ABTS: EC_50_ = 29.3 ± 0.4 µM (Ascorbic acid EC_50_ = 23.3 ± 0.2 µM) [58]
35	DPPH: EC_50_ > 250 µM (Ascorbic acid EC_50_ = 23.3 ± 0.2 µM) [58]; EC_50_ = 20.5 µM (α-Tocopherol EC_50_ = 27.1 µM) [62] ABTS: EC_50_ = 175.4 ± 3.9 µM (Ascorbic acid EC_50_ = 23.3 ± 0.2 µM) [58]
36	DPPH: EC_50_ > 250 µM (Ascorbic acid EC_50_ = 23.3 ± 0.2 µM) [58] ABTS: EC_50_ = 45.8 ± 1.7 µM (Ascorbic acid EC_50_ = 23.3 ± 0.2 µM) [58]

DPPH, 2,2-diphenyl-1-picrylhydrazyl; ABTS, 2,2′-azino-bis(3-ethylbenzothiazoline-6-sulphonic acid); EC_50_, Effective concentration for half maximal activity; NBT, nitroblue tetrazolium; BHA, Butylated hydroxyanisole; ^a^, Values obtained by conversion of original units.

A glance at the results indicated in Table 3 shows that the most used positive control is ascorbic acid, a commercially available potent antioxidant with remarkable beneficial effects in human health [83] and the recommended standard for the DPPH antioxidant assay [80]. Another important assumption is that out of the 26 compounds assayed, 13 had higher activity than the tested positive controls being in some cases nearly three times more active than ascorbic acid. These interesting results can corroborate the plant traditional use.

Following the DPPH assay results, it can be disclosed that the most active compound (2.8-fold more active than ascorbic acid) is adenodimerin A (**25**), a new proanthocyanidin type A recently isolated from *Morella adenophora* roots [58]. Compound **14**, a myricanol derivative, and compounds **20**, **23** and **26**, flavonoid derivatives, showed DPPH scavenging activity lower than **25** and also lower than α-tocopherol and BHA, other commercially available antioxidant references. However, the authors [62,69] did not present the associated errors and this lowers the credibility in their results. Regarding the error associated to EC_50_ values, it is negatively surprising that, beyond the cases where it is not shown [62,69], several other results showed standard deviation higher than 5% (compounds **1** and **9** in DPPH assay and compounds **9**, **20** and **25** in ABTS assay) and even higher than 10% (compound **3** in DPPH assay and compounds **2** and **11** in ABTS assay). Such high margins of error make the results unreliable and therefore diminish their scientific interest. Authors, reviewers and editors must be more and more attentive to this so that published works have the greatest rigor and impact.

We were confronted with another difficulty that result from the significantly fluctuating EC_50_ values, as an example we can highlight the EC_50_ results for compound **12** in two DPPH assays, moreover the authors [58,62] used the same method, only the positive control is different, and this cannot explain the very high variation.

Table 3 analyses of the DPPH assay data also allowed interesting conclusions about diarylheptanoid antioxidant action: (a) diarylheptanoid derivatives are less active than flavonols, only compounds **3** and **14**, exhibit EC_50_ below 20 µM and are more active than ascorbic acid and α-tocopherol respectively; (b) a hydroxyl group at carbon C-11 instead of a carbonyl do not improve the activity (e.g., compounds **1** and **10**); (c) an extra hydroxyl group at carbon C-5 is also irrelevant (e.g., compounds **1** and **2**); (d) on the other hand the loss of a methyl group (e.g., compounds **2** and **3**) causes a strong increase in antioxidant effect; (e) it seems that the presence of a sugar moiety, the type of sugar and its localization, also interfere with the antioxidant activity (e.g., compounds **10** to **14**).

Nevertheless the results obtained with several flavonols in the NBT assays allowed some interesting conclusions: (a) the presence of an extra hydroxyl group at C-3′ increases the activity (e.g., compounds **18** and **19**); (b) the presence of sugar derivative substituent at C-3 also increases the activity (e.g., compounds **17**, **18**, **20** and **23**).

From our literature survey and resumed in Table 3 it is noteworthy that the antioxidant evaluation against the most common ROS species are unusual.

Although oleanane-type compounds are recognized as important scaffolds [76] the derivatives found in *Morella*/*Myrica* genus were almost inactive in the antioxidant evaluations performed.

Our literature research herein presented and discussed revealed that *in vivo* antioxidant studies involving compounds isolated from *Myrica*/*Morella* genus were nor reported, naturally due to the difficulties inherent to the *in vivo* methodologies. However, the *in vitro* studies do not reflect the compound’s actual antioxidant activity. After their consumption, compounds can be metabolized and lose activity, but above all can generate unwanted metabolites that can be toxic. So greater effort from the scientific community to use *in vivo* methodologies as well as investigations of toxicity and side effects are highly recommended.

It can also be noted that other species of these genera should be studied and more attention to their fruits is also needed.

Recent works enhance the application of the *Morella* and *Myrica* species, as supplement and/or nutraceutical were not herein discussed because they are beyond the scope of this review; in fact they deal with extract mixtures and not with pure isolated compounds [45,84,85].

### 4.2. Anti-Inflammatory Activity

As referred at the beginning of this work, a high intracellular ROS level may activate various ROS-sensitive signaling pathways and promote inflammatory gene expression in a very large number of clinical situations involving very different symptomatology. In the inflammatory process there are several mediators, some of which have been used as the prime targets to find new anti-inflammatory agents. The most common are: (a) the pro-inflammatory cytokines such as IL-1β, IL-6, TNF-α, mediators of immune response that contribute for the activation and amplification of the inflammatory response pathway initiated with Th1 cells [86,87]; (b) nitric oxide, a mediator similar to neurotransmitters in the neuronal system, which can be produced by inducible nitric oxide synthase stimulated by cytokines and bacterial pathogens [88,89]; (c) PGE_2_ (prostaglandin E2), a known inflammatory mediator in chronic diseases, being a major compound in late/chronic inflammation and is also a common pharmaceutical target for both steroidal and non-steroidal anti-inflammatory drugs [90].

Compounds isolated from *Myrica* and *Morella* species were assayed for their anti-inflammatory activity and effect on some of the most common inflammatory markers, the most relevant data is gathered in Table 4. Again we excluded some results due to the fact that authors do not report IC_50_ values and inhibition percentage do not provide reliable information.

**Table 4:** Anti-inflammatory a7ctivities of isolated compounds from *Myrica* and *Morella* species.

Compound	Anti-Inflammatory Activity (Positive Control Used) ^a^	Reference
1	IC_50_ (iNOS) = 1.0 ± 0.1 µM ( *N*ῳ-nitro-l-arginine IC_50_ = 39.5 ± 2.7 µM Aminoguanidine IC_50_ = 22.2 ± 3.6 µM)	[58]
3	IC_50_ (iNOS) = 13.0 ± 0.9 µM ( *N*ῳ-nitro-l-arginine IC_50_ = 39.5 ± 2.7 µM Aminoguanidine IC_50_ = 22.2 ± 3.6 µM)	[58]
	IC_50_ (NO) = 63.51 µM ( *N*-monomethyl-l-arginine IC_50_ = 64.24 µM) *	[63]
4	IC_50_ (NO) = 30.19 µM ( *N*-monomethyl-l-arginine IC_50_ = 64.24 µM)	[63]
5	IC_50_ (iNOS) = 46.9 ± 3.1 µM ( *N*ῳ-nitro-l-arginine IC_50_ = 39.5 ± 2.7 µM Aminoguanidine IC_50_ = 22.2 ± 3.6 µM)	[58]
6	IC_50_ (NO) = 23 µM ( *N*ῳ-nitro-l-arginine IC_50_ = 28 µM)	[71]
7	IC_50_ (NO) = 46.18 µM ( *N*-monomethyl-l-arginine IC_50_ = 64.24 µM)	[63]
10	IC_50_ (iNOS) = 7.5 ± 2.7 µM ( *N*ῳ-nitro-l-arginine IC_50_ = 39.5 ± 2.7 µM Aminoguanidine IC_50_ = 22.2 ± 3.6 µM)	[58]
15	IC_50_ (NO) = 45.32 µM ( *N*-monomethyl-l-arginine IC_50_ = 64.24 µM)	[63]
16	IC_50_ (TNF-α) = 20.1 ± 2.14 µM (PDTC IC_50_ = 16.8 ± 2.13 µM; Quercetin IC_50_ = 13.6 ± 0.81 µM); IC_50_ (IL-1β) = 22.9 ± 0.75 µM (PDTC IC_50_ = 18.0 ± 1.74 µM; Quercetin IC_50_ = 16.9 ± 0.34 µM) IC_50_ (IL-6) = 22.7 ± 1.61 µM (PDTC IC_50_ = 16.8 ± 2.40 µM Quercetin IC_50_ = 16.8 ± 0.13 µM)	[67]
17	IC_50_ (TNF-α) = 12.90 ± 0.84 µM (PDTC IC_50_ = 25.32 ± 0.51 µM) IC_50_ (IL-1β) = 18.06 ± 3.16 µM (PDTC IC_50_ = 23.61 ± 2.17 µM) IC_50_ (IL-6) = 7.69 ± 2.14 µM (PDTC IC_50_ = 21.41 ± 1.69 µM)	[68]
18	IC_50_ (TNF-α) = 8.65 ± 1.62 µM (PDTC IC_50_ = 25.32 ± 0.51 µM) IC_50_ (IL-1β) = 18.97 ± 2.15 µM (PDTC IC_50_ = 23.61 ± 2.17 µM) IC_50_ (IL-6) = 13.14 ± 0.44 µM (PDTC IC_50_ = 21.41 ± 1.69 µM)	[68]
19	IC_50_ (TNF-α) = 1.55 ± 1.15 µM (PDTC IC_50_ = 25.32 ± 0.51 µM) IC_50_ (IL-1β) = 17.84 ± 1.56 µM (PDTC IC_50_ = 23.61 ± 2.17 µM) IC_50_ (IL-6) = 8.63 ± 2.14 µM (PDTC IC_50_ = 21.41 ± 1.69 µM)	[68]
20	IC_50_ (TNF-α) = 65.21 ± 3.11 µM (PDTC IC_50_ = 25.32 ± 0.51 µM)	[68]
	IC_50_ (IL-1β) = 22.81 ± 2.51 µM (PDTC IC_50_ = 23.61 ± 2.17 µM) IC_50_ (IL-6) = 23.65 ± 6.14 µM (PDTC IC_50_ = 21.41 ± 1.69 µM) IC_50_ (NO) = 99 µM ( *N*ῳ-nitro-l-arginine IC_50_ = 28 µM)	[71]
21	IC_50_ (TNF-α) = 19.9 ± 2.45 µM (PDTC IC_50_ = 16.8 ± 2.13 µM Quercetin IC_50_ = 13.6 ± 0.81 µM) IC_50_ (IL-1β) = 20.2 ± 1.42 µM (PDTC IC_50_ = 18.0 ± 1.74 µM; Quercetin IC_50_ = 16.9 ± 0.34 µM) IC_50_ (IL-6) = 22.2 ± 1.14 µM (PDTC IC_50_ = 16.8 ± 2.40 µM Quercetin IC_50_ = 16.8 ± 0.13 µM)	[67]
22	IC_50_ (TNF-α) = 20.1 ± 2.14 µM (PDTC IC_50_ = 16.8 ± 2.13 µM Quercetin IC_50_ = 13.6 ± 0.81 µM) IC_50_ (IL-1β) = 22.9 ± 0.75 µM (PDTC IC_50_ = 18.0 ± 1.74 µM Quercetin IC_50_ = 16.9 ± 0.34 µM) IC_50_ (IL-6) = 22.7 ± 1.61 µM (PDTC IC_50_ = 16.8 ± 2.40 µM Quercetin IC_50_ = 16.8 ± 0.13 µM)	[67]
23	IC_50_ (iNOS) = 30.9 ± 2.1 µM ( *N*ῳ-nitro-l-arginine IC_50_ = 39.5 ± 2.7 µM Aminoguanidine IC_50_ = 22.2 ± 3.6 µM)	[58]
	IC_50_ (TNF-α) = 25.20 ± 0.54 µM (PDTC IC_50_ = 25.32 ± 0.51 µM) IC_50_ (IL-1β) = 25.04 ± 0.48 µM (PDTC IC_50_ = 23.61 ± 2.17 µM) IC_50_ (IL-6) = 13.41 ± 1.81 µM (PDTC IC_50_ = 21.41 ± 1.69 µM)	[68]
	IC_50_ (NO) > 100 µM	[71]
24	IC_50_ (iNOS) = 45.4 ± 0.89 µM ( *N*ῳ-nitro-l-arginine IC_50_ = 39.5 ± 2.7 µM Aminoguanidine IC_50_ = 22.2 ± 3.6 µM)	[58]
28	IC_50_ (NO) = 30.19 µM ( *N*-monomethyl-l-arginine IC_50_ = 64.24 µM)	[63]
31	IC_50_ (NO) = 24 µM ( *N*ῳ-nitro-l-arginine IC_50_ = 28 µM	[71]
32	IC_50_ (NO) between 3–10 µM ( *N*ῳ-nitro-l-arginine IC_50_ = 28 µM)	[71]
33	IC_50_ (iNOS) = 39.5 ± 2.7 µM ( *N*ῳ-nitro-l-arginine IC_50_ = 39.5 ± 2.7 µM Aminoguanidine IC_50_ = 22.2 ± 3.6 µM)	[58]
35	IC_50_ (iNOS) = 18.1 ± 1.5 µM ( *N*ῳ-nitro-l-arginine IC_50_ = 39.5 ± 2.7 µM Aminoguanidine IC_50_ = 22.2 ± 3.6 µM)	[58]
	IC_50_ (NO) = 23 µM ( *N*ῳ-nitro-l-arginine IC_50_ = 28 µM)	[71]

^a^, iNOS, Inducible nitric oxide synthase; TNF-α, Tumour necrosis factor α; IL, Interleucine; NO, nitric oxide production in lipopolysaccharide-stimulated RAW 264.7 cells; PDTC, Pyrrolidine dithiocarbamate; IC_50_, Inhibitory concentration for half maximal enzyme activity.

The anti-inflammatory potential of *Myrica*/*Morella* species seems to be effective, since from the 43 assays whose results are shown in Table 4, 13 exhibited higher activities than the positive control tested which is quite remarkable. The diarylheptanoids myricanone **1** and myricanol **10**, are very active iNOS inhibitors, since they are, respectively, 20 and four times more active than the most active control tested (aminoguanidine IC_50_ = 22.2 µM), while the flavonols myricetin 3-*O*-(2-*O*-galloyl)-β-D-galactopyranoside **18** and quercetin 3-*O*-(2-*O*-galloyl)-β-D-galactopyranoside derivatives **19** are able to reduce significantly the release of pro-inflammatory cytokines (TNF-α, IL-1β, and IL-6).

Despite the high anti-inflammatory potential revealed by the results shown in Table 4, some of them have unusually high standard deviation values (>10% activity value) and their higher activity should be considered with caution. For instance we can indicate three particularly surprising cases, the inhibitory effect of compound **17** on IL-6 (7.69 ± 2.14 µM) [68], of compound **10** on iNOS (7.5 ± 2.7 µM) [58] and of compound **19** on TNF-α (1.55 ± 1.15 µM) [68], where errors are displayed from 28% to 74%. Also results without any statistical evaluation were reported [71]. This limits the impact of these results, and again indicates that results should always be evaluated critically.

In view of the structure/activity relationship, it is noteworthy that Kim *et al.* [67] also tested the inhibitory effect on TNF-α, IL-1β and IL-6 of the compounds **16**, **21** and **22** aglycones, however their results did not reveal significant changes in the activity. These results are important to demonstrate that the 3′-*O*-sulfate group in a flavonoid structure can be used to improve the compound solubility in water, without affecting its anti-inflammatory action. As far as we are aware, these authors were also the only ones that simultaneously measured RAW 264.7 cell viability at a concentration greater than the IC_50_ value. In fact, only with this methodology is it possible to conclude that anti-inflammatory effect is not due to cytotoxicity of the compounds tested.

The results also showed that compound 20 is less active to inhibit the TNF-α and IL-6 cytokines than its 3-*O*-rhamnoside derivative 23 and even less effective than derivatives 17 and 18. On the other hand, compound 19, whose structure differs from compound 18 only in an additional 3′-OH group, reduces the release of pro-inflammatory cytokines TNF-α and IL-6 more than any of the foregoing compounds. These facts suggest that: (a) the non-free 3-OH group is an important structural scaffold to show inhibitory activity on cytokines TNF-α and IL-6; (b) once more the presence of a galloyl group, seems to have a positive effect on the activity.

In Table 4 are included results where the compound tested is not more active than the positive control, meaning that the tested compounds are not good enough. But their activity can provide some scientific support for the traditional uses reported in Table 1.

Again we can conclude that more in depth studies are lacking, for instance toxicity evaluations and *in vivo* studies.

## 5. Conclusions

Secondary metabolites with unusual structures and exhibiting antioxidant and anti-inflammatory activities, high enough to capture the attention of researchers and to be considered as potential drug leads, were isolated from *Myrica*/*Morella* species; some of the traditional medicine uses of these species are adequately justified. It is nonetheless curious that, while the fruits of *Morella* and *Myrica* species are edible, and therefore commercially quite valuable, most of the relevant studies on antioxidant and anti-inflammatory activities of the compounds tested were isolated from bark, leaves and roots.

Clearly, much has been done, but much remains to do. From the biological evaluation point of view: (a) toxicity testing is lacking; (b) more specifically in-depth studies on the mechanisms of action are needed; (c) *in vivo* evaluations are necessary; (d) standardization of the methodologies; and (e) more compounds should be tested to elaborate the more detail structure-activity relationships. From the chemical point of view: (a) more species should be phytochemicaly analyzed; and (b) more detailed analysis in the compounds structure characterization is necessary, since for example some compounds are reported and tested without their stereogenic centers properly elucidated.

This review hopes to raise awareness and drive interest for further research on these genera with minds set on ensuring the quality of traditional uses and maybe turning them into pharmacological alternatives to the ones that already exist on the market.
